# Tobacco Smoking Behaviors in Cancer Survivors: The Mediation Effect of Personality and Emotional Intelligence

**DOI:** 10.3390/curroncol29120742

**Published:** 2022-12-02

**Authors:** Ilaria Durosini, Marianna Masiero, Chiara Casini, Gabriella Pravettoni

**Affiliations:** 1 Applied Research Division for Cognitive and Psychological Science, IEO, European Institute of Oncology IRCCS, 20141 Milan, Italy; 2 Department of Oncology and Hemato-Oncology, University of Milan, 20123 Milan, Italy

**Keywords:** decision-making, personality, smoking, emotional intelligence, cancer, cancer survivors

## Abstract

The smoking behaviour of patients following a cancer diagnosis is a critical risk factor for several physical diseases; it can increase the risk of second primary tumors and lower cancer treatment efficacy. Despite this, a great number of survivors continue to smoke after the diagnosis. This observational, cross-sectional on-line study aimed to assess the relationship between the impact of cancer diagnosis on survivors and their smoking behavior, and whether emotional intelligence and personality might mediate this relationship. Ninety-four Italian survivors completed a set of questionnaires: *Big Five Inventory*; *Fagerström Test for Nicotine Dependence*; *Impact of Event Scale; Brief Emotional Intelligence Scale*. The results obtained from the mediation analyses highlighted that the indirect effect on the relationship between the psychological impact of the diagnosis and smoking behaviors was partially mediated by neuroticism (Intrusion: 95% CI [0.00; 0.11]; Avoidance: 95% CI [0.00; 0.18]). Additionally, the data suggested that the relationship between the psychological impact of the diagnosis and smoking behaviors was partially mediated by the utilization of emotions dimension of emotional intelligence (Intrusion: 95% CI [0.00; 0.10]; Avoidance: 95% CI [0.00; 0.22]). Overall, this study suggests the importance of designing interventions to support smoking interruption based on the “*mapping*” of individual needs and emotional regulation strategies.

## 1. Introduction

As reported by the International Scientific Community around the World, tobacco smoking behavior is a critical risk factor for several respiratory, cardiovascular, and oncological diseases. Considering the cancer disease occurrence, 30% of all cancer deaths and nearly 90% of lung cancer deaths are caused by tobacco cigarette smoking [1]. In addition, 60% of oncological patients are seemingly current smokers, recent quitters, or former smokers [1]. This late datum is alarming for oncologists and other healthcare professionals, considering that continued cigarette smoking has a central role in the recovery process of cancer patients during acute, extended, and permanent survivorship [2,3]. Indeed, cigarette smoking is associated with increased treatment toxicity, a higher risk of treatment failure, a higher incidence of second tobacco-related malignancies, and, finally, a shorter period of survival [1,4,5]. Evidence has reported that smoking could lead to alterations in the biology of cancer cells, leading to treatment resistance and, as a consequence, increasing mortality [6]. This alarming trend requires systematic prevention actions in order to understand the psychological and cognitive mechanisms behind continued tobacco cigarette smoking during the cancer continuum (from early diagnosis to extended survivorship) [7,8].

### 1.1. Smoking Behaviors and Personality

Over the years, a growing body of evidence has pointed out the role of *personality* and affect regulation as two of the foremost predictors of smoking behaviors. Eysenck and colleagues conducted original studies on the relationship between personality and smoking in 1960, which stressed that smokers are more extroverted, less rigid, and more neurotic compared with non-smokers [9]. This could be related to the fact that individuals with high neuroticism are expected to experience more stress and anxiety than others [10], and this aspect may negatively impact immune functioning [11]. As a means to cope with these negative emotions, people with high neuroticism tend to adopt unhealthy behaviors, such as smoking [12,13,14]. However, some authors pointed out that a specific kind of neuroticism could also lead to some benefits for health under certain circumstances [15]. This could depend on how people deal with their emotions (e.g., worry and anxiety). In this line, Weston and Jackson [16] highlighted the concept of *healthy neuriticism*, defined as a “positive response to stress and uncertainty” (p. 61). This kind of personality trait, on the contrary of traditional neuroticism, acts as a trait that supports or discourages unhealthy behaviors, improving vigilance and concerns about somatic symptoms and treatments [16,17]. Coherently, healthy neuroticism may potentially be beneficial for non-smoking behavior, improving people’s capacity to deal with their negative affects [16]. This may particularly occur when high levels of neuroticism traits are paired with high levels of conscientiousness traits. As highlighted by Weston and Jackson [16], neurotic traits allow people to be concerned about their health [15], whereas conscientiousness allows people to act in order to manage these concerns, for example, by changing their behaviors in order to promote their health and adhere to healthcare recommendations [18,19]. In this line, individuals with high neuroticism and consciousness are more prone to reduce the number of cigarettes smoked in a day or avoid smoking behaviors [20]. In fact, Terraciano and Costa [13] highlighted that current smokers tend to show the highest combination of high neuroticism and low conscientiousness and the lowest combination of high neuroticism and high conscientiousness. These aspects suggest that personality traits, such as the combination of neuroticism and conscientiousness, may play a role in smoking behaviors, improving the risk of cigarette smoking initiation and the maintenance/cessation of this unhealthy behavior [13].

Additionally, studies based on Cloninger’s pharmacogenetic model of personality [21,22,23] reported that chronic smokers scored higher than non-smokers on questions related to harm avoidance and novelty seeking. In line with these results, Etter [24] highlighted similar findings, observing that smokers display a higher level of novelty seeking and harm avoidance and have lower persistence and poorer self-direction, when compared to former smokers. Overall, Munafò and colleagues [25] have suggested that these results might be interpreted according to a twofold perspective. First, a high level of extraversion in smokers might be explained by a high need for sociability, which might be related to a dopaminergic alteration induced by nicotine inhalation contained in cigarettes. Second, high scores on neuroticism might be explained by the smoker’s need to control negative emotions (e.g., anxiety, fear, irritability, frustration, and depression) or, conversely, by tobacco-induced serotonergic inhibition [25].

### 1.2. Smoking and the Management of Emotions

Even if personality might be used to understand addictive behaviors, other psychological mechanisms are involved, such as affect regulation [26]. Affect regulation refers to any attempt to cope with negative emotions and mood states using behavioral, cognitive, and environmental-change methods. For example, in coherence with the self-medication hypothesis [27], smokers might use cigarettes as a strategy to cope with stress, anxiety, depression, and more general negative affects [28,29]. As suggested by Tomkins [30], “*smoking can be learned to relieve any negative affect and to evoke any positive affect. So, we may learn to pick up a cigarette to make us feel less afraid, less angry, less ashamed, less disgusted. We may also learn to pick up a cigarette to give us a positive affective lift of excitement*.” (p. 18). This aspect might be particularly important for people facing an oncological diagnosis, in which negative physical and psychological long-term side effects could affect patients’ quality of life [31]. Due to the need for regular treatments and monitoring, patients and survivors have to make essential changes in their life plans and lifestyles [32,33,34]. Coherently, from a psychological point of view, the cancer experience might cause psychological distress, anxiety, worry, fear, and depression [35], which can have an additional impact on their health [36]. Cancer survivors may also experience fear of cancer recurrence, that is the persistent fear that the tumor may return in their life; this could lead to depressive episodes, anxiety, and fluctuations in their motivations to manage their health [37]. Further, in smoker patients, these negative emotions can be exacerbated and frequently associated with feelings of guilt and shame. For example, lung cancer patients with a smoking history tend to blame themselves for their disease. These negative emotions are associated with a higher level of internalized stigma, depression, and anxiety [38]. In this vein, smoker patients may use cigarettes to regulate their mood and emotions, and to cope with psychological distress [39], in turn reinforcing smoking and reducing the likelihood of interrupting. Recently, the role of emotional intelligence (EI) has been highlighted as a possible psychological determinant supporting different phases of the smoker’s career (onset, interruption, and relapse) [40,41,42]. According to Salovey and Mayer [43], EI is the personal ability to monitor, perceive, and express one’s own and others’ emotions, discriminate among themes, and use this information to manage personal thinking and behaviors. Subsequently, Goleman [44] described the construct of EI as the personal ability to regulate and recognize emotions in themselves and others [45]. The construct of EI was at the center of a controversy in which several authors debated its conceptualization as a structure of ability [46] or as a personality trait [47]. Taking into consideration the conceptualization of EI as a trait, five dimensions could be described as core components of EI: (i) the ability to recognize emotions in themselves and identify aspects that could change these emotions; (ii) the ability to interpret emotions in others; (iii) perceptions of control and regulating emotions; (iv) the ability to foster positive feelings in other people; and (v) the ability to use positive emotions for problem-solving [48,49]. González-Yubero and colleagues [42] stated that these dimensions are significant predictors of alcohol, tobacco, and illegal substances. Accruing evidence on cigarette smoking conveyed that lower levels of EI are related to smoking frequency, earlier initial smoking age, and the worst awareness of the adverse costs of smoking [41]. Limonero and colleagues [50] found that cigarette smokers have a lower value in emotional repair. Overall, people unable to repair their emotional state are more at risk of starting smoking. Other studies found similar results, observing that smokers characterized by high emotional clarity and repair had a reduced likelihood of relapse [51]. In addition, a higher level of EI seems to affect comprehension and awareness of the danger associated with cigarettes [40]. Overall, studies suggested that smokers might use cigarette smoking to ameliorate their emotional regulation deficit. Although the relationship between EI and smoking behavior appears well documented in the healthy population, little is known about this relationship in the cancer population. To our knowledge, no studies have been performed matching the evaluation of personality, smoking behaviors, and EI in a sample of cancer survivors.

### 1.3. Aim and Hypothesis

The current observational, cross-sectional study aimed to assess the possible relationship between the impact of diagnosis on cancer survivors and smoking behavior and whether EI and personality traits might mediate this relationship.

We hypothesized that personality and EI may be the cause of continued smoking in cancer survivors. In particular, smokers with low EI levels used cigarettes to face the traumatic experience of cancer diagnosis and its associated negative emotions. Further, we argue that smoker survivors are characterized by specific personality traits (neuroticism and extraversion) that might explain their continued smoking.

## 2. Materials and Methods

### 2.1. Procedure and Measures

The study employed an observational, cross-sectional, and prospective design with a convenience sampling method. All of the participants were recruited through social networks, groups of people who received an oncological diagnosis and personal contacts. Their anonymity and the confidentiality of the data were guaranteed. Participation in the study was voluntary; at each moment, participants could decide to withdraw. As a part of the recruitment process, participants were required to fulfill the following inclusion criteria: (i) participants must be adults (≥18 years old); (ii) have received a diagnosis of cancer; and (iii) must be Italian speakers. The survey was conducted from January to July 2022. The participants were invited to sign an online informed consent and to complete a battery of questionnaires using the QUALTRICS software. The study followed the principles stated in the Declaration of Helsinki (59th WMA General Assembly, Seoul, 2008).

The following questionnaires were administered:

*Brief Emotional Intelligence Scale* (BEIS-10) [49]: The BEIS-10 is a 10-item self-report questionnaire that assesses EI in adults. This scale investigates the individual dispositions in exploring and managing one’s own and other’s emotions and feelings through five dimensions: *appraisal of one’s own emotions* (i.e., ability to recognize one’s emotions and to identify factors that could modify them); *appraisal of others’ emotions* (i.e., ability to interpret the emotions of others); *regulation of one’s own emotions* (i.e., ability to control and regulate one’s emotions); *regulation of others’ emotions* (i.e., the ability to foster positive feelings in other people); and *utilization of emotions* (i.e., people’s ability to use one’s positive emotions for problem-solving) [48]. In addition, participants report their extent of agreement for each item according to a five-point Likert scale, ranging between “totally disagree” and “totally agree”. The BEIS-10 was recently validated in the Italian context, showing adequate reliability (α = 0.73) [49]. In this study, Cronbach’s alpha coefficient is equal to 0.84 for the total scale.

*Big Five Inventory* (BFI) [52]: The BFI is a 44-item self-report questionnaire on a 5-point scale, from strongly disagree to strongly agree, that assesses the five traits/dimensions of personality: *Openness to Experience, Conscientiousness, Extraversion, Agreeableness,* and *Neuroticism*. The Italian validation of this scale showed adequate reliability, with the Cronbach’s alpha coefficients ranging between 0.69 (*Agreeableness*) and 0.83 (*Conscientiousness*) [53]. In this study, the scale showed adequate reliability, with the Cronbach’s alpha coefficients ranging between 0.65 and 0.81 (specifically, *Openness to Experience*: α = 0.77, *Conscientiousness*: α = 0.75, *Extraversion*: α = 0.80, *Agreeableness*: α = 0.65, *Neuroticism*: α = 0.81).

*Impact of event scale* (IES) [54,55]: The IES explores, through 15 items, the impact of traumatic experiences. The IES includes two of the most commonly reported categories of experiences in response to stressful events: *intrusion* (that is, related to intrusively experienced ideas, feelings, images, or dreams) and *avoidance* (that is, related to consciously recognized avoidance of certain ideas, situations, or feelings). This study used the Italian version of the scale [55]. Participants were invited to complete the scale referring to their experience of cancer. The scale showed good reliability in Italian validation, with a Cronbach’s alpha equal to 0.84 for *intrusion* and 0.71 for *avoidance* [55]. In the same line, in this study, the IES showed Cronbach’s alpha coefficients equal to 0.84 and 0.57 for *intrusion* and *avoidance* dimensions, respectively.

Lastly, the participants were asked to declare their cigarette smoking behaviors and were included into two groups: (1) people who actually smoked or have smoked tobacco cigarettes in the past and (2) people who were never smokers, people who had never smoked in their life. Smokers are defined as people who regularly smoked at the enrollment time and/or have smoked in the past, for at least one year. On the other hand, never smokers are participants that had never smoked in their life. This sample also involves people who have tried a cigarette (*experiential phase*), but have not continued to smoke.

The participants included in the first group were also invited to complete the *Fagerström Test for Nicotine Dependence* [56], a brief six-item self-report questionnaire assessing nicotine dependence that conceptualizes dependence through behavioral and physiological symptoms. The original validation reported a Cronbach’s alpha of 0.61 [56], while the Cronbach’s alpha coefficient in the Italian validation was 0.55 [57]. Further, a set of ad hoc items were used to evaluate starting age and the number of daily cigarettes. In the present sample, women in the smoking group started using tobacco at a younger age (*M* = 18.98, *SD* = 5.30). Among people who actually smoke, women smoked around ten cigarettes per day (*SD* = 6.93), and the smoking dependence determined by the Fagerström Test for Nicotine Dependence showed a low dependence index (*M* = 2.18; *SD* = 1.68).

### 2.2. Data Analysis

The descriptive statistics were computed for all of the socio-demographic variables and the questionnaire scores. Further, the *t*-Student analyses were computed to explore the differences in the impact of traumatic experiences (*intrusion* vs. *avoidance* subscale) between smokers and non-smokers. Additionally, we conducted mediation analysis to assess the possible mediated role of EI and personality in the relationship between smoking behaviors and the impact of the cancer diagnosis. Mediation analyses were conducted to assess any possible mediation effect by EI (*appraisal of one’s own emotions*; *appraisal of others’ emotions*; *regulation of one’s own emotions*; *regulation of others’ emotions*; and *utilization of emotions*) and personality traits (*Extraversion; Agreeableness; Conscientiousness; Neuroticism; Openness to experience*) in the relationship of the psychological impact of the diagnosis (*Intrusion; Avoidance*) and smoking status (Smokers vs. Non-Smokers). In the same line, the mediation analyses were computed to assess the possible mediation role of personality (*Openness to Experience, Conscientiousness, Extraversion, Agreeableness,* and *Neuroticism*) in the relationship between the psychological impact of the oncological diagnosis and smoking/no-smoking behaviors.

Thus, EI and personality traits were used individually as mediators, and analyses were conducted with the PROCESS procedure developed for SPSS (Hayes v3.4—Model 4) [58]. This boot-strapping technique is used to reveal any possible mediational effect of EI and personality traits. All the results were obtained based on 5000 bootstrapped samples. Statistical significance occurs if the 95% bias-corrected confidence intervals for the indirect effect do not include zero [59].

## 3. Results

### 3.1. Participants

Ninety-four Italian women who previously received an oncological diagnosis participated in this study. Most of them had a history of breast cancer (*n* = 74, 74.7%). The age of participants ranged between 27 and 77 years old (*M_age_* = 52.06, *SD_age_* = 8.26), and a great number of participants (*n*= 64, 68.1%) were in a relationship or engaged. The level of education was medium-high, and 50 participants had a university degree or more (53.2%). In order to assess psychological differences related to smoking attitudes, participants were divided into two groups according to their history of smoking. The first group included 51 participants who actually smoked or have smoked in the past (“Smokers”; age range: 26–77 years old, *M_age_* = 52.49, *SD_age_* = 7.82). They received the diagnosis of cancer at an average of 5.39 years ago (*SD*= 5.18) and the majority of them were in treatment (66.66%), especially with hormonoterapia (41.18%).

In contrast, 43 participants who did not smoke (“Non-Smokers”; age range: 34–77 years old, *M_age_* = 51.56, *SD_age_* = 8.81) were included in the second group. They received the diagnosis of cancer at an average of 6.12 years ago (*SD*= 5.25) and the majority of them are in treatment (62.79%), especially with hormonoterapia (44.19%).

No difference emerged in the medium age (*F*(1) = 0.295, *p* = 0.59), in the level of education (*X*^2^(3) = 3.56; *p* = 0.31), and in employment (*X*^2^(3) = 6.87, *p* = 0.76) across the groups. Table 1 reports all the details of the sample. All the participants consented to the study and completed the questionnaire online.

### 3.2. The Impact of the Diagnosis and Smoking Behaviors: Differences among Groups

In order to conduct mediation analyses, we explore, through the *t*-Student test, differences in the impact of traumatic experiences (*intrusion* vs. *avoidance* subscale) between groups. The data highlighted no differences in the psychological impact of the diagnosis in smokers and non-smokers (Table 2).

### 3.3. Mediation Analyses

#### 3.3.1. The Mediation Effect of Emotional Intelligence on Traumatic Experiences (IES)

The direct effect of the intrusive dimension (IES-Intrusion) on smoking status (*β* = 0.026, *s.e.* = 0.046, *p* = 0.565, 95% CI [−0.064; 0.117]) was not statistically significant. In the same line, the mediation analyses highlighted that the path (direct effect) from the intrusively experienced feelings about cancer to *Appraisal of own emotions* (*β* = –0.057, *s.e.* = 0.033, *p* = 0.086, 95% CI [−0.12; 0.01]), *Appraisal of others’ emotions* (*β* = −0.041, *s.e.* = 0.033, *p* = 0.212, 95% CI [−0.11; 0.02]), *Regulation of others’ emotions* (*β* = –0.029, *s.e.* = 0.036, *p* = 0.421, 95% CI [−0.10; 0.04]) was not statistically significant. Meanwhile, the direct effect of IES-Intrusion to *Regulation of own emotions* (*β* = −0.056, *s.e.* = 0.029, *p* = 0.05, 95% CI [−0.11; 0.00]) and *Utilization of emotions* (*β* = −0.052, *s.e.* = 0.027, *p* = 0.05, 95% CI [−0.10; 0.00]) was statistically significant.

**Figure 1:**
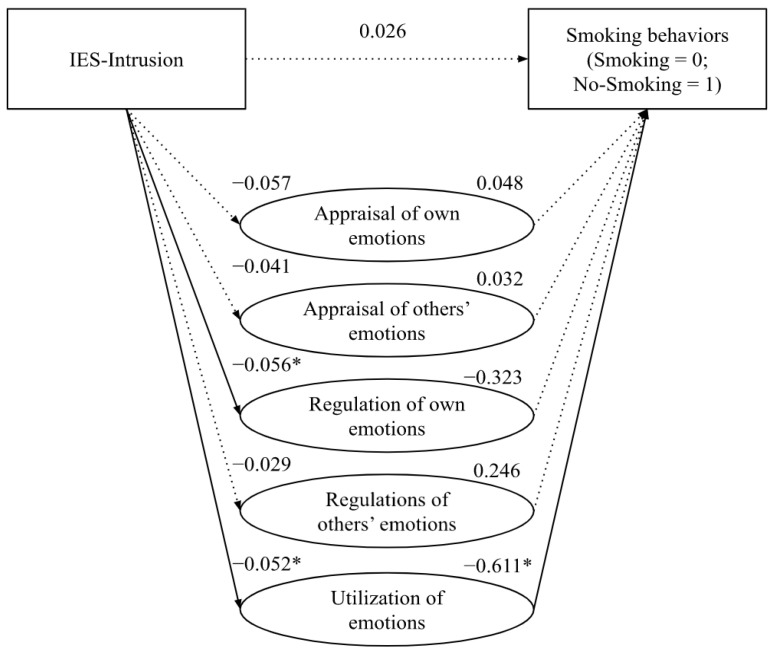
Partial mediation effect of emotional intelligence on the relationship between intrusion and smoking behaviors. *Note*. * *p* < 0.05.

The path (direct effect) from the *Appraisal of own emotions* (*β* = 0.048, *s.e.* = 0.179, *p* = 0.789, 95% CI [−0.302; 0.398]), *Appraisal of others’ emotions* (*β* = 0.032, *s.e.* = 0.173, *p* = 0.853, 95% CI [−0.307; 0.371]), *Regulation of own emotions* (*β* = −0.323, *s.e.* = 0.221, *p* = 0.139, 95% CI [−0.759; 0.107]), *Regulation of others’ emotions* (*β* = 0.246, *s.e.* = 0.164, *p* = 0.133, 95% CI [−0.075; 0.567]) to smoking status was not statistically significant. In contrast, the direct effect of the *Utilization of emotions* on smoking status was negative and statistically significant (*β* = −0.611, *s.e.* = 0.256, *p* < 0.05, 95% CI [−1.112; −0.110]) (see Figure 1).

**Figure 2:**
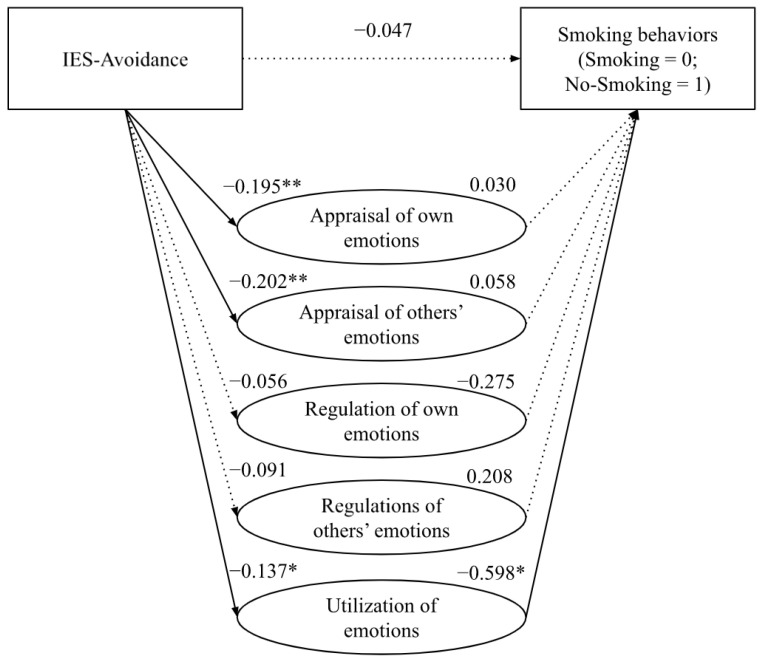
Partial mediation effect of emotional intelligence on the relationship between avoidance and smoking behaviors. *Note*. ** *p* < 0.01; * *p* < 0.05.

The direct effect of the conscious avoidance of certain aspects relating to the impact of cancer experience (IES-Avoidance) on smoking behaviors was not statistically significant (*β* = −0.047, *s.e.* = 0.105, *p* = 0.652, 95% CI [−0.253; 0.158]). In the same line, the mediation analyses highlighted that the path (direct effect) from the tendency to avoid certain ideas or feelings related to the cancer experience to *Appraisal of own emotions* (*β* = −0.195, *s.e.* = 0.072, *p* < 0.01, 95% CI [−0.339; −0.052]), *Appraisal of others’ emotions* (*β* = −0.202, *s.e.* = 0.071, *p* < 0.01, 95% CI [−0.343; −0.061]), and *Utilization of emotions* (*β* = −0.137, *s.e.* = 0.060, *p* < 0.05, 95% CI [−0.255; −0.018]) was negative and statistically significant. In contrast, the direct effect form IES-Avoidance and *Regulation of own emotions* (*β* = −0.056, *s.e.* = 0.066, *p* = 0.401, 95% CI [−0.187; 0.076]) and *Regulation of others’ emotions* (*β* = −0.091, *s.e.* = 0.079, *p* = 0.251, 95% CI [−0.248; 0.066]) was not statistically significant. The path (direct effect) from the *Appraisal of own emotions* (*β* = 0.030, *s.e.* = 0.176, *p* = 0.865, 95% CI [−0.315; 0.375]), *Appraisal of others’ emotions* (*β* = 0.058, *s.e*. = 0.170, *p* = 0.732, 95% CI [−0.275; 0.392]), *Regulation of own emotions* (*β* = −0.275, *s.e.* = 0.217, *p* = 0.205, 95%CI [−0.699; 0.150]), *Regulation of others’ emotions* (*β* = 0.208, *s.e.* = 0.156, *p* = 0.182, 95% CI [−0.098; 0.514]) to smoking behaviors was not statistically significant. However, the direct effect of the *Utilization of emotions* on smoking behaviors was negative and statistically significant (*β* = −0.598, *s.e.* = 0.252, *p* < 0.05, 95% CI [−1.091; −0.104]). The data suggested that the relationship between intrusively experienced ideas or feelings about cancer and smoking behaviors was partially mediated by the *utilization of emotions dimension of emotional intelligence* (Indirect Effect = 0.082, *s.e.* = 0.058, 95% CI [0.00; 0.22]; see Figure 2, Table 3).

#### 3.3.2. The Mediation Effect of Personality Traits on Traumatic Experiences (IES)

**Figure 3:**
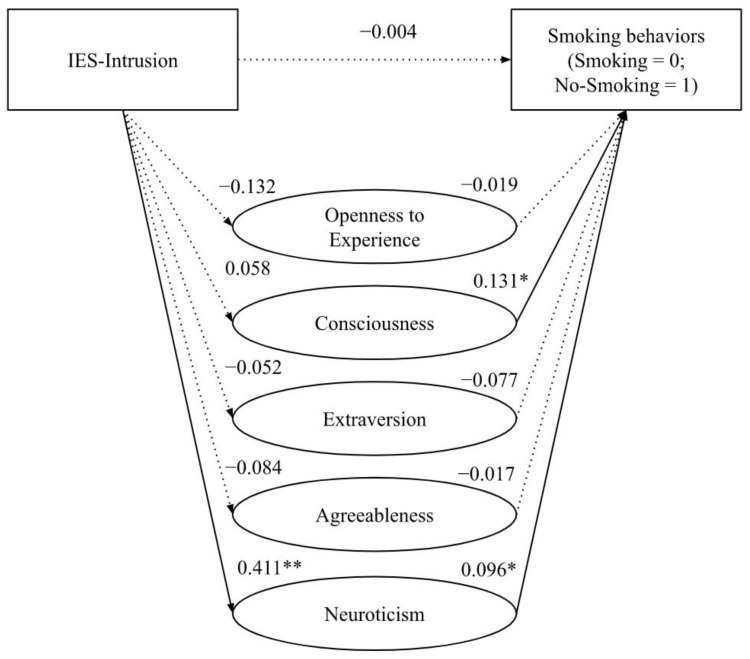
Partial mediation effect of personality on the relationship between intrusion and smoking behaviors. *Note*. ** *p* < 0.01; * *p* < 0.05.

Additionally, we explored whether smoking status is a function of intrusively experienced ideas or feelings about cancer (IES-Intrusion) and personality traits. The path (direct effect) from the Intrusion dimension of IES to smoking behaviors is not statistically significant (*β* = −0.004, *s.e.* = 0.046, *p* = 0.937, 95% CI [−0.086; 0.093]). The direct effect of the intrusive thoughts about cancer on openness to experience, conscientiousness, extraversion, and agreeableness was not statistically significant (*Openness*: *β* = −0.132, *s.e.* = 0.115, *p* = 0.253, 95% CI [−0.361; 0.096]; *Consciousness*: *β* = 0.058, *s.e.* = 0.088, *p* = 0.509, 95% CI [−0.116; 0.232]; *Extraversion*: *β* = −0.052, *s.e.* = 0.119, *p* = 0.666, 95% CI [−0.289; 0.186]; *Agreeableness*: *β* = −0.084, *s.e.* = 0.079, *p* = 0.293, 95% CI [−0.241; 0.074]). In contrast, the direct effect of intrusive thoughts about cancer on *Neuroticism* was positive and statistically significant (*β* = 0.411, *s.e.* = 0.117, *p* < 0.01, 95% CI [0.179; 0.642]), indicating that people who showed higher intrusive thoughts related to their cancer experience showed a higher score in neuroticism traits. Similarly, the direct effect of *Openness to Experience, Extraversion,* and *Agreeableness* to smoking behaviors were not statistically significant (*Openness*: *β* = −0.019, *s.e.* = 0.046, *p* = 0.682, 95% CI [−0.108; 0.071]; *Extraversion*: *β* = −0.077, *s.e*. = 0.049, *p* = 0.112, 95% CI [−0.172; 0.018]; *Agreeableness*: *β* = −0.017, *s.e.* = 0.065, *p* = 0.792, 95% CI [−0.145; 0.111]). In contrast, the direct effect of *Consciousness* (*β* = 0.131, *s.e.* = 0.067, *p* < 0.05, 95%CI [0.000; 0.262]) and *Neuroticism* (*β* = 0.096, *s.e.* = 0.050, *p* = 0.05, 95% CI [0.00; 0.193]) on smoking behaviors was positive and statistically significant, suggesting that people who scored higher in *Conscientiousness* and *Neuroticism* are more likely to smoke after cancer. In summary, the data suggested that the relationship between intrusively experienced feelings about cancer and smoking behaviors was not partially mediated by *Openness to Experience, Conscientiousness, Extraversion, and Agreeableness* but was partially mediated by *Neuroticism* (Indirect Effect = 0.039, *s.e.* = 0.030, 95% CI [0.00; 0.11]; see Figure 3, Table 3).

**Figure 4:**
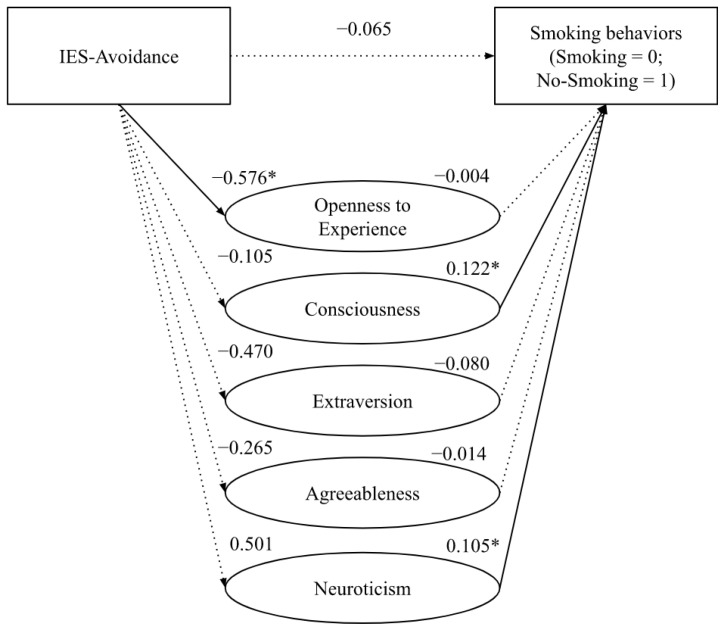
Partial mediation effect of personality on the relationship between avoidance and smoking behaviors. *Note*: * *p* < 0.05.

Lastly, we assessed whether a person’s smoking status is a function of avoidance towards thoughts related to the cancer experience (IES-Avoidance) and the five personality traits. The direct effect from the IES-Avoidance to smoking behaviors is not statistically significant (*β* = −0.065, *s.e.* = 0.098, *p* = 0.508, 95% CI [−0.257; 0.127]). The direct effect of Avoidance on *Conscientiousness*, *Extraversion*, *Agreeableness*, and *Neuroticism* were not statistically significant (*Consciousness*: *β* = −0.105, *s.e.* = 0.194, *p* = 0.590, 95% CI [−0.383; 0.092]; *Extraversion*: *β* = −0.470, *s.e.* = 0.258, *p* = 0.072, 95% CI [−0.983; 0.043]; *Agreeableness*: *β* = −0.265, *s.e.* = 0.173, *p* = 0.129, 95% CI [−0.610; 0.079]; *Neuroticism*: *β* = 0.501, *s.e.* = 0.270, *p* = 0.067, 95% CI [−0.035; 1.037]). In contrast, the direct effect of Avoidance on *Openness to Experience* (*β* = −0.576, *s.e.* = 0.262, *p* < 0.05, 95% CI [−1.10; −0.056]) was negative and statistically significant, indicating that people who tend to be avoidant of their feelings relating to their cancer diagnosis showed a lower score in *Openness to Experience*. The direct effect of *Openness to Experience*, *Extraversion*, and *Agreeableness* to smoking behaviors were not statistically significant (*Openness*: *β* = −0.004, *s.e.* = 0.043, *p* = 0.919, 95% CI [−0.089; 0.081]; *Extraversion*: *β* = −0.080, *s.e.* = 0.049, *p* = 0.099, 95% CI [−0.176; 0.015]; *Agreeableness*: *β* = −0.014, *s.e.* = 0.065, *p* = 0.830, 95%CI [−0.141; 0.113]). Whereas, the direct effect of *Consciousness* (*β* = 0.122, *s.e.* = 0.064, *p* = 0.05, 95% CI [0.00; 0.247]) and *Neuroticism* (*β* = 0.105, *s.e.* = 0.047, *p* < 0.05, 95% CI [0.013; 0.198]) on smoking behaviors was positive and statistically significant. Overall, the data highlighted that the indirect effect on the relationship between the avoidance of feelings about cancer and smoking behaviors was partially mediated by *Neuroticism* (Indirect Effect = 0.053, *s.e.* = 0.050, 95% CI [0.00; 0.18]; Figure 4, Table 3).

## 4. Discussion

The results retrieved by our study highlighted that the psychological impact of cancer diagnosis is not directly related to smoking behaviors in cancer survivors, but other psychological determinants act as a proxy. This aspect means that the psychological impact of cancer, which could lead people to have intrusively experienced feelings or to adopt an avoidant attitude related to certain ideas or situations, does not necessarily play a direct role in the adoption and the maintenance of tobacco cigarette smoking behavior during all phases of cancer survivorship. In coherence with our hypothesis, our results suggested a crucial role played by *EI* and *personality* as potential mediators of *continued smoking* during survivorship’s trajectory. In particular, the data suggested that cancer survivors with a higher ability to use positive emotions to foster problem-solving are more likely to reduce intrusively experienced feelings about cancer and to reduce avoiding feelings related to their diagnosis. Additionally, survivors who showed high neuroticism are more likely to experience intrusive feelings related to their cancer diagnosis, while open survivors are more likely to reduce the tendency to avoid some thoughts related to their oncological experience. When attempting a mediational model, a dimension of EI, namely the ability to use positive emotions for problem-solving, mediated the association between the psychological impact of cancer and smoking behaviors.

In the same line, the intrusively experienced ideas of cancer appear as a significant predictor of a refusal of cigarette smoking only with the mediation of the ability to use personal emotions to solve their problems. Overall, the tendency to avoid some aspects related to the diagnosis or the tendency to experience intrusive feelings related to cancer are not related to the smoking behavior per se, but are related to the individuals’ ability to use personal emotions to resolve problems in their life. We argue that survivors might adopt tobacco cigarette smoking in order to use their positive emotions for problem solving to allow them to reduce their avoidant thoughts related to the cancer and to reduce the difficulty of remaining in contact with their thoughts related to the disease. These results are coherent with the evidence collected on the association between smoking and emotions, suggesting that smokers use cigarettes to manage their emotions [27,30].

Concerning personality traits, a key result concerns the positive relationship between neuroticism, conscientiousness and survivors who never smoked. Additionally, when attempting a mediational model, neuroticism mediated the association between the psychological impact of the diagnosis and tobacco cigarette smoking behaviors. In particular, the consciously recognized avoidance of certain feelings related to cancer appears as a significant predictor of smoking behavior, only with mediation by neuroticism. Similarly, intrusive cancer memory appears as a significant predictor of smoking behavior only with the mediation of neuroticism. This means that the tendency to avoid some aspects related to the diagnosis or the tendency to experience intrusively feelings are not related to the smoking behaviour per se, but are related to the individual’s tendency to experience irritability and anxiety. Similarly, people who showed high neuroticism tended to avoid tobacco use when avoidant feelings were present. When attempting a mediational model, the presence of intrusive feelings related to cancer appears as a significant predictor of smoking behavior with mediation by neuroticism. People who showed high neuroticism tended to avoid tobacco use when intrusive feelings were present. Intrusive feelings about cancer might act as a proxy for fear and worry, such as protective emotions favoring smoking interruption.

These late results are coherent with existing studies that have highlighted the existence of an *healthy neuroticism* as a “positive response to stress and uncertainty” [16] (p. 61). The authors suggested that neuroticism might act as a trait supporting or discouraging smoking behavior in coherence with the type of emotion regulation of negative effects such as anxiety, worry, and fear of cancer recurrence. Neurotic survivors might focus their attention on their body and symptoms, increasing the detection of the body signals suggesting a possible disease occurrence. This type of behavior increases the possibility of adopting healthy behaviors [16]. Other studies suggest that the combination of neuroticism and conscientiousness traits in smokers affects both frequency of smoking and the amount of daily cigarette [13,16,20].

### Limitations and Future Research

A possible limitation of this study is the fact that the participants involved in this research received different cancer diagnoses. Although this aspect might be helpful in understanding the impact of oncological diagnosis on smoking behavior, this prevented us from attaining a deeper understanding of the impact of a specific kind of diagnosis on the explored relationship. For example, future studies could involve survivors who received a specific diagnosis of cancer, such as lung cancer, to understand the specific role of psychological dynamics and traits on smoking behaviors. Another limitation of this study is that the participants included in the study showed a low dependence index of smoking (as determined by the Fagerström Test for Nicotine Dependence index: *M* = 2.18; *SD* = 1.68) and a medium/high level of emotional intelligence (*M* = 37.86, *SD* = 5.95). Additionally, all the participants were females with a history of cancer. Future studies may include people with different levels of smoking dependence to form a deeper understanding of the decision to quit or not quit smoking after oncological diagnosis. Additionally, future research may explore the proposed model among people of both sexes and in different chronic populations. The cross-sectional nature of the design allows us to be cautious in hypothesizing causal relationships between the explored variables because the data collection could expose us to a defensive stance or false responses because of sensitive topics. Future studies must explore the role of contingency factors as possible contributors to the explored relationship. For example, by assessing how dark triad traits, which tend to be negatively related to health behaviors and the rumination of life experiences, may contribute to explaining the explored relationships better. This aspect would be consistent with the previous literature that suggested that dark triad traits were negatively related to healthy behaviors [60].

## 5. Conclusions

The study sheds light on the possible relationship between the traumatic experience of cancer diagnosis and continued smoking behaviors during active and extended survivorship, considering the key mediation role of personality traits and EI. Our results suggest that specific personality traits and individual strategies to manage emotions might act as inner determinants of continued smoking in cancer survivors to face the traumatic experience of a cancer diagnosis. In particular, two milestones seem to be suggested by our study. The first is related to the neuroticism that might increase in cancer survivors’ negative emotions related with their past diagnosis, supporting the adoption of health behaviors, such as no-smoking behavior. The second is the role of positive emotions for problem solving used by smoker survivors to reduce their avoidant thoughts related to the cancer; this, in turn, reduces the difficulty of remaining in contact with their thoughts related to the disease.

Overall, the results highlight the importance of designing interventions to support smoking interruption based on the “*mapping*” of individual needs [61,62] and emotional regulation strategies. We argue that antismoking psychological interventions for cancer survivors should increase awareness concerning inner emotional mechanisms and individual strategies used to face the stress of life events, such as cancer diagnosis and its sequels. As suggested, the mapping might also support high-risk patients and survivors in dealing with the shame and stigma connected to the disease, increasing their active role in the care pathway [61].

## Figures and Tables

**Table 1:** Descriptive data of participants.

	Smokers (*n* = 51)		Non-Smokers (*n* = 43)			
Descriptive Data	*n*	%	*n*	%	*X^2^* (df)	*p*
*Tumor*					7.62 (8)	0.471
Breast	41	85.4	37	86.0		
Bowel	1	2.1	1	2.3		
Lung	0	0	1	2.3		
Pancreas	1	2.1	0	0		
Kidney	1	2.1	0	0		
Uterus	4	8.3	2	4.7		
Skin	0	0	1	2.3		
Bones	0	0	1	2.3		
*Level of education*					3.561 (3)	0.313
Secondary school	5	9.8	2	4.7		
High school	22	43.1	15	34.9		
Master Degree	17	33.3	14	32.6		
Post University Degree	7	13.7	12	27.9		
Employment					6.87 (3)	0.076
Unemployed	13	25.5	6	14.0		
White collar	26	51	24	55.8		
Blue collar	8	15.7	3	7.0		
Self-employed/director	4	7.8	10	23.3		

**Table 2:** Differences among groups in the impact of traumatic experiences.

	Smokers	Non-Smokers	*t* (df)	*p*
IES-Intrusion	17.43 (4.96)	18.83 (5.98)	−1.20 (86)	0.232
IES-Avoidance	7.38 (2.13)	7.47 (2.74)	−0.160 (88)	0.874

**Table 3:** Bootstrapped indirect effects of the association between the impact of cancer, EI, personality traits, and smoking behaviors.

	Effect (β)	SE	95% CI
*IES-Intrusion*			
Appraisal of own emotions	−0.003	0.017	[−0.04; 0.03]
Appraisal of others’ emotions	−0.001	0.013	[−0.03; 0.03]
Regulation of own emotions	0.018	0.019	[−0.01; 0.06]
Regulation of others’ emotions	0.007	0.014	[−0.04; 0.02]
Utilization of emotions	0.032	0.029	[0.00; 0.10]
*IES-Avoidance*			
Appraisal of own emotions	−0.006	0.048	[−0.11; 0.09]
Appraisal of others’ emotions	−0.012	0.047	[−0.12; 0.08]
Regulation of own emotions	0.015	0.040	[−0.06; 0.11]
Regulation of others’ emotions	−0.019	0.029	[−0.09; 0.03]
Utilization of emotions	0.082	0.058	[0.00; 0.22]
*IES-Intrusion*			
Openness to Experience	0.003	0.011	[−0.02; 0.03]
Conscientiousness	0.008	0.018	[−0.02; 0.05]
Extraversion	0.004	0.013	[−0.03; 0.03]
Agreeableness	0.001	0.009	[−0.02; 0.02]
Neuroticism	0.039	0.030	[0.00; 0.11]
*IES-Avoidance*			
Openness to Experience	0.003	0.034	[−0.07; 0.08]
Conscientiousness	−0.013	0.041	[−0.10; 0.07]
Extraversion	0.038	0.041	[−0.02; 0.14]
Agreeableness	0.004	0.025	[−0.04; 0.07]
Neuroticism	0.053	0.050	[0.00; 0.18]

## Data Availability

The data presented in this study are available on request from the corresponding author.
